# Feeding Our Microbiota: Stimulation of the Immune/Semiochemical System and the Potential Amelioration of Non-Communicable Diseases

**DOI:** 10.3390/life12081197

**Published:** 2022-08-05

**Authors:** David Smith, Sohan Jheeta, Hannya V. Fuentes, Miryam Palacios-Pérez

**Affiliations:** 1 Network of Researchers on the Chemical Evolution of Life (NoRCEL), Leeds LS7 3RB, UK; 2 Instituto de Investigaciones Biomédicas, Universidad Nacional Autónoma de México (UNAM), Mexico City 04510, Mexico

**Keywords:** microbiota, microbiome, microbiota–gut–brain axis, non-communicable diseases, prebiotics, probiotics, semiochemicals, ingestible sensor

## Abstract

Non-communicable diseases are those conditions to which causative infectious agents cannot readily be assigned. It is increasingly likely that at least some of these conditions are due to the breakdown of the previously mutualistic intestinal microbiota under the influence of a polluted, biocide-rich, environment. Following the mid-20th century African studies of Denis Burkitt, the environmental cause of conditions such as obesity has been ascribed to the absence of sufficient fibre in the modern diet, however in itself that is insufficient to explain the parallel rise of problems with both the immune system and of mental health. Conversely, Burkitt himself noted that the Maasai, a cattle herding people, remained healthy even with their relatively low intake of dietary fibre. Interestingly, however, Burkitt also emphasised that levels of non-communicable disease within a population rose as faecal weight decreased significantly, to about one third of the levels found in healthy populations. Accordingly, a more cogent explanation for all the available facts is that the fully functioning, adequately diverse microbiome, communicating through what has been termed the microbiota–gut–brain axis, helps to control the passage of food through the digestive tract to provide itself with the nutrition it needs. The method of communication is via the production of semiochemicals, interkingdom signalling molecules, potentially including dopamine. In turn, the microbiome aids the immune system of both adult and, most importantly, the neonate. In this article we consider the role of probiotics and prebiotics, including fermented foods and dietary fibre, in the stimulation of the immune system and of semiochemical production in the gut lumen. Finally, we reprise our suggestion of an ingestible sensor, calibrated to the detection of such semiochemicals, to assess both the effectiveness of individual microbiomes and methods of amelioration of the associated non-communicable diseases.

## 1. Introduction: The Unstoppable Rise of Obesity

By the late 20th century, it was clear that dietary and behavioural treatments for the control of obesity were failing [1] and yet, interestingly, the accumulated evidence pointed to the idea that people were following the recommendations of the time and were, indeed, eating less than had been normal in earlier years, at least in Britain [2]. By the early 21st century, experiments involving heavy isotope labelled water confirmed that levels of physical activity of people were similar in distinct parts of the world, were about what could be expected for similar-sized mammals and, moreover, had not undergone notable change since the 1980s [3]. As no new ideas were available at the time, there was little to do except bemoan the failure of these “biology and genetics” approaches [4], to recognise that the field is full of unsupported assumptions, indeed plain guesses [5], and to count the substantial cost of the obesity crisis [6]. Needless to say, levels of obesity continue to rise across the world [7], along with a worryingly rapid drop in strength, especially noticeable in schoolchildren, as reported both in England [8], and in Slovenia [9].

However, alongside the essentially thermodynamic studies of the late 20th century, there was an increasing interest in the bacteria inhabiting our intestine. One observation caused an immediate stir, that obesity could be transferred as if it were an infectious disease, at least among the microbiota of mice [10]. Unfortunately, this apparent lead turned out not to be useful, and it remains possible to study aspects of obesity without any reference to the microbiome whatsoever [11]. Alongside these deliberations, however, the existence of the microbiota–gut–brain axis was noted when its degradation became associated with obesity [12]. Interestingly, Denis Burkitt strongly suggested early on that elevated levels of dietary fibre reduced the levels of non-communicable disease, including obesity (see discussion, below) [13]; and although his suggestions were not fully borne out in practice, his dietary fibre hypothesis has been given renewed emphasis by observations about the production of the energy-supplying short chain fatty acids acetate, propionate and butyrate (SCFAs) within the microbiome under the influence of high-fibre diets [14]. Although these leads are still being pursued, Burkitt nevertheless also noted that the cattle-rearing Maasai did not eat a high-fibre diet and yet remained healthy, in his day at least [13].

By contrast, our own work suggests that the mutualistic microbiome behaves as a combination of immune and messenger chemical (semiochemical) systems stemming from the Precambrian Vertebrata. The presence of non-communicable disease further suggests that it has been damaged by the use of caesarean section and by biocides, including heavy metal pollution [15], and that the damage is increased from one generation to the next in an ongoing “snowball effect” following transfer of a malfunctioning microbiome in the apparently accidental process that we have termed *maternal microbial inheritance* [16].

Note that, while the term “microbiota” is a consortium of microorganisms including bacteria and archaea, fungi (mycobiome), and viruses (virome) that have evolved to live cooperatively in each ecosystem, for example, inside human body, the term microbiome includes both the symbiotic microorganisms and their genes with beneficial characteristics; microeukaryotes are present in the microbiome and are assumed to play a significant role but are not adequately defined as yet. However, we use the two terms microbiota and microbiome interchangeably, primarily to avoid repetition and to smooth the flow of discussion. Overall, the focus of this article is on the microbiome as a “black box”, in which the key point is the output of the microbiota, rather than the complexity of the system within the intestine itself.

## 2. Health versus Pollution: The Degraded Microbiome

The earliest metagenomic analyses of the human microbiome was carried out on two reportedly healthy adults using the 16S ribosomal DNA sequences, illustrating the diversity of microbial form and function that we now take for granted [17], obsoleting the idea that all microbes are malignant *per se*. Interestingly, of course, we now know that greater microbial diversity is associated with health [18,19], bringing into question the concept of what exactly constitutes a “healthy adult” in the first place, and also of what a human being is constituted, because human attributes combine with the result of the metabolism of millions of microbes [17] in the mouth, throat, nose, skin, vagina, saliva, and intestines [20]. e focused on guts because there is located the largest sensory organ of the human body [21], and the bidirectional communication between the intestines and the brain, the microbiota–gut–brain axis, possibly arose long time ago during the Precambrian Vertebrata [15].

The Yanomami, a people living in the Venezuelan–Brazilian Amazon, are among the healthiest in terms of their blood lipid profiles, with no sign of obesity, at least until they leave their communities and enter what can loosely be termed the “modern world” [22], suggesting that “westernisation affects human microbiome diversity” [23]. Likewise, the Tsimane, from the Bolivian Amazon, not only show low levels of cardiovascular disease [24], but also little dementia, despite their high systemic inflammation [25]. Lest people think that this is unique to South America, the Hadza, a people from Tanzania, were similarly healthy and, moreover, were found not to carry any strains of Bifidobacteria [26], a group of microorganisms currently held to confer health benefits [27], albeit not very successfully [28].

In the mid-20th century, Denis Burkitt studied the health gap between what he called “Modern Western Civilization” on the one hand, and traditional African societies on the other, finding a host of non-communicable diseases among the former that were simply absent from the latter [13]. Having discerned an environmental cause, and knowing nothing of the microbiome, he then went on to surmise that the low levels of fibre in modern foods stood for a form of dietary deficiency. However, not only could he not connect the inflammatory forms of non-communicable disease with dietary fibre, but he also reported that the cattle-rearing Maasai (Masai in his day) were free from such disease, despite their relatively low fibre, modern-type diet [13]. By contrast, the epidemiology of many non-communicable conditions is more consistent with selective poisoning of the microbiome by the pollution associated with industrialisation, leading to disease following a deficiency of microbiome function [29]. It is likely that this microbiome–body relationship was first developed in the Precambrian Vertebrata, and that the disconnect between microbiome and gut is due specifically to heavy metal poisoning [15]. Accordingly, it follows that no one born in a polluted environment can be considered safe from non-communicable disease [15]; by following this argument, there is no absolute definition for the term “healthy adult”. In terms of the potential for disease, the mantra must be “guilty until proven innocent”.

## 3. Genes versus Environment: Maternal Microbial Inheritance

While it has always been realised that the action of our genes must, somehow, be modified by factors within our environment, more attention has been paid to the former, with its readable sequences and theoretical opportunities for modification, but the precise significance of the term “environment” has been left in abeyance. Regarding obesity, Claude Bouchard and his team performed an experiment in the 1980s in which twelve pairs of young adult genetically identical twins were over-fed by 1000 kcal per day above their normal baseline food intake. Prior to the experiment, neither the twins themselves, nor their parents, showed any sign of excessive adiposity or specific lipid-related diseases. Over 100 days, all the twin pairs had gained weight, but each pair, though similar within themselves, were very different from one another [30], these results were presented in terms of their genetics. At about the same time, David Barker was developing his hypothesis by stating that the nature of non-communicable diseases in adults, including schizophrenia, could be traced back to their childhood, or even into the womb itself [31]. Although much speculated on [32], and economically literate [33], this “fetal origins hypothesis” has never been fully accepted. Our suggestion is that the microbiome of the mother represents a part of the overall environment in which the individual genetic code of the child operates. Epigenetic mechanisms for temporary deactivation of protein synthesis have been known since the mid-20th century [34], but more recently, there have been investigations into the existence of heritable epigenetic mechanisms [35], but not without debating the viability of the available evidence [36]. Of course, such questions would be answered if the microbiome inherited from the mother were capable of epigenetic control over aspects of the development of the child. As yet, however, there is little known about the ability of bacteria to exert control over genetic processes [37], let alone any skills obtained by the uptake of mobile genetic elements or, indeed, those conferred by unicellular eukaryotes [15].

As with Burkitt’s findings discussed above, nobody knew about the microbiome, nor that it was being transferred from mother to child by the seemingly accidental process of maternal microbial inheritance [16]. Of course, twins are born at the same time from the same mother so, accordingly, the outcome of Bouchard’s experiment [30] would have been affected by both their somatic gene sequence and, also, the genes belonging to their microbiota. While this accounts for the intra-pair similarity, in a sense the microbiome stands for a factor associated with the environment, as the inter-pair variability shows a significant prior influence on the mother, such as may be associated with antibiotic treatment, for example. Similarly, in principle this accounts for Barker’s epidemiological observations about the source of adult disease, albeit in the modified form of an “infant origins hypothesis” [31]. Interestingly, the presence of significant differences *within* genetically identical twin pairs may, perhaps, be attributable to differential microbial contamination following delivery by caesarean section [38], or simply differences in their adaptive immune system [39].

A similar argument applies to the findings of David Strachan, in which immune system malfunctioning in the infant will lead to future problems such as asthma and hay fever [40]. While Strachan’s original “hygiene hypothesis” has been followed up by Rook and co-workers, still no trace of an *external* immune system training agent has been found [41]. Nevertheless, the possibility remains that the fully functioning microbiome carries an *internal* agent which enables the calibration of the immune system against the microbial environment of the mother. Such an agent could represent a so-far hypothetical microbial equivalent of the dendritic cells of our own human immune system [42], which we have previously described as microbial sentinel cells [43], and suggested that they may stem from the Precambrian Animalia [15].

## 4. The Mutualistic Microbiome: External Microbes and Their Antigens

Of course, all foods are closely associated with potentially pathogenic microbial contamination and, while cleaning and disinfection may reduce the microbial load [44], even cooking still leaves certain genetic sequences more or less intact [45]. Presumably these microbial fragments include plasmids and other mobile genetic elements that can become incorporated into the functioning microbiome and, in turn, can be passed on to the neonate in preparation for the microbial world it will soon inhabit as an independent entity [15]. While weaning a child onto solid foods is usually achieved relatively easily, an adult eating uncooked food in a foreign environment may suffer from dramatic consequences [46]. Although the causative agents of traveller’s diarrhoea are the commonly called pathogens, of course they have no effect on people originally brought up in those countries. Needless to say, as these conditions are unpleasant but rarely life-threatening, to our knowledge there has been little research on the possibility of adults themselves becoming immune to this whole new set of microbes. Instead, assuming the worse effects are avoided, it is interesting to speculate as to whether this probiotic-like immune stimulation of the microbiota–gut–brain axis can actually contribute to the enjoyment of the overall experience of foreign travel.

Significantly, however, it is worth noting that most of the microbiome-related work has been conducted on the degraded microbiome, as described above, and focuses virtually exclusively on the prokaryote constituents. Although the relative absence of work on the potentially critical microeukaryote components of the microbiome has been noted [47,48], there are exceptions, such as a study on the early development of the human mycobiome [49]. Of course, the ability of *Toxoplasma gondii* to influence the brain of a mammal illustrates the potential organising ability of unicellular eukaryotes [50]. Although, as with *T. gondii*, such species are almost invariably treated as parasites, it is noteworthy that *Blastocystis* species are commonly observed in apparently healthy individuals [51] and can pass between different animal species relatively easily [52]. Nevertheless, if one role of hypothetical microbial sentinel cells is to seek out novel antigens to effectively calibrate the immune system of the child compared to that faced by the mother, it could be that such cells are simply no longer present in populations chronically exposed to heavy metal pollution [15].

Although there is currently no reason to assume that traveller’s diarrhoea is altered by microbiome-function deficiency disease, it is worth mentioning that the whole spectrum of allergic and autoimmune diseases could come within its range. Significantly, the first comprehensive description of what we now know as seasonal allergic rhinitis was by John Bostock in the early 19th century [53], at about the time when Burkitt mentioned descriptions of obese people becoming common in art and literature [13]. Thinking that he was on to something important, Bostock continued his search for people with unambiguous symptoms, eventually uncovering a grand total of 28 from all over the British Isles. Compare this with the situation in Britain in recent times, with a report of 21% of schoolchildren taking medication in 2005 [54]. While hay fever is itself trivial, of course there is a relationship with food allergy [55] and atopic disease in general [56]. Although he could not have understood its significance, Bostock stressed that the sufferers were “all from the highest ranks of society” [57], and it is probable that their mothers were using heavy metal-based cosmetics [58], thus condemning their children to the sort of diseases we are familiar with today [29]. While the symptoms of hay fever are unmistakable, the same cannot be said for mental illness and, when faced with such problems among rich people in 19th century Vienna, Sigmund Freud had no precedent to fall back on. Although psychoanalysis eventually became respectable [59], of course it was never fully accepted. Indeed, it is telling that Burkitt’s otherwise comprehensive review never mentioned mental health at all [13].

## 5. The Mutualistic Microbiome: The Immune/Semiochemical Complex

Rather than consider all the interactions between the different microbes both with one another and with the gut wall, it is more valuable to consider the microbiome as a “black box”, an object whose internal workings are a still an intricate mystery but whose output is significant [60]. During the initial investigations, it was noted that so-called germ-free mice exhibited an unnatural stress response [61]. As noted in the Introduction, in due course the term microbiota–gut–brain axis has become recognised as a significant component of the healthy body due, at least in part, to its association with obesity [12]; on the other side, individuals with anorexia nervosa manifest a reduction in microbiota diversity and there is a significant association with depression, anxiety, and lack of appetite [62]. It seems that there are two classes of significant chemical output from the microbiome: molecules associated with energy supply on the one hand, and interkingdom signalling molecules, semiochemicals, on the other. Note that, by this definition, any bodily hormones or related chemicals sending signals to modify the behaviour of the microbiome are also semiochemicals.

In reporting his African studies, Burkitt emphasised that a substantially greater faecal weight, indeed as much as three-fold, was associated with the absence of non-communicable disease [13]. Interestingly, it has been reported that greater stool microbial diversity has been associated with a higher level of gut motility, presumably implying that signals from the microbiome (psycho-active substances such as dopamine, serotonin, and catecholamines) improve peristalsis [63].

Signalling molecules have been described as being produced in the gut lumen, including the catechol dopamine [64], which has a role in controlling aspects of the immune system [65]. Dopamine generated within the brain has also been shown to affect systemic glucose production, possibly as a part of the overall microbiota–gut–brain axis [66]. In a similar fashion, while the microbiota produces both energy-related B-vitamins and the short chain fatty acids (SCFAs) acetate, propionate, and butyrate [67], these latter compounds contribute to the production of serotonin [68], as well as aspects of the immune system [69]. Accordingly, all these molecules can be classed as part of the dual immune system/semiochemical output of the mutualistic microbiome [70]. Interestingly, it seems that the ability to synthesise these key semiochemicals could have been passed on from bacteria to animal cells by horizontal gene transfer at some stage during their evolution [71].

## 6. Breaking the Contract: Dysbiosis as Microbiome Failure

While the name “dysbiosis” seems an excellent shorthand for a malfunctioning microbiome, the term is imprecisely defined [72]. Likewise, Harald Brüssow has pointed out that there is a need for more investigation into causal relationships between specific bacterial commensals and disease states, within a “sound ecological and evolutionary” rationale [73]. Our own suggestion is that there is essentially no connection between specific bacteria and disease, rather it is the inability of the depleted microbiome to support mobile genetic elements that is the critical factor [16]. In support of this thesis, we further suggest that the rationale for the microbiome is to add the flexibility of horizontal gene transfer at the microbial level to the relative stability of multicellular evolution by the inheritance of acquired characteristics, thus combining the benefits of operating both above and below what has been called the Darwinian threshold [74]. Note that the failure of the microbiome is at an evolutionary level, albeit that this failure is reflected in a myriad of seemingly different conditions [75]. Of course, this is closely analogous to the holobiont concept pioneered by Lynn Margulis [76,77,78]. Nevertheless, it is both the nature and the timing of the non-communicable diseases resulting from the breakdown of mutualism that supplies the best sign as to the role of the fully functioning microbiome prior to its degradation.

**Figure 1:**
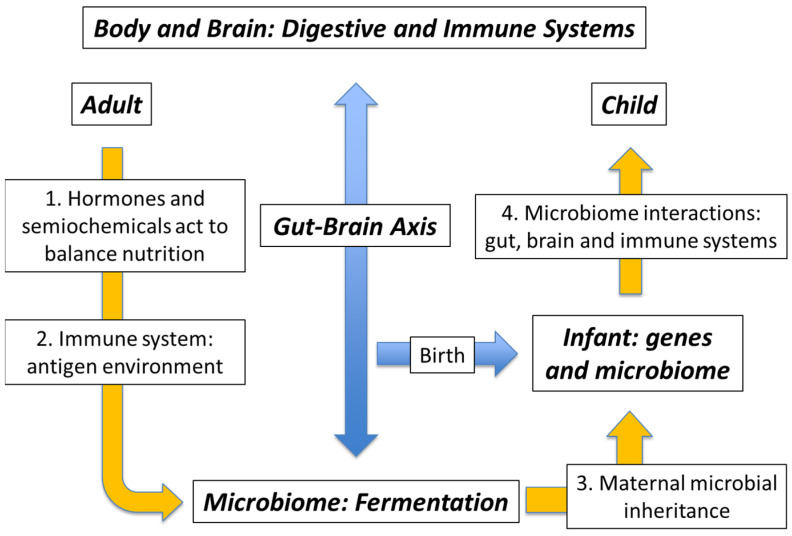
Semiochemical/immune complex: Adult and child.

In the light of Barker’s original observation of the “fetal and infant origins of adult disease”, it seems likely that both the immune system and the microbiota–gut–brain axis start to develop immediately after birth [75], and that any lack of microbial function will have a negative impact on the eventual health of the individual, to a greater or lesser extent [29]. The concept of mutualism implies two parallel interactions that benefit both components. Thus, while the microbiome guest calibrates the immune system of the neonate against the microbial environment of its host mother, the adult must, in turn, respond by supplying nutrition to its microbial guest ready for the next generation [15]. It is important to note, however, that this host–guest relationship must be sufficiently flexible to cope with the expected events of life: accident and illness; famine and, of course, the special conditions of pregnancy [79]. Although the details are not yet clear, it is possible to imagine the microbiome providing a steady level of semiochemical-delivered demand for nutrition, balanced against a variable hormone-delivered demand from the body. If so, any inclement conditions may lead to an increase in hormone-delivered demand followed by microbiome shutdown until conditions improve. Our suggestion is that the mutualistic microbiome operates across the generations as illustrated in Figure 1 [15].

Left hand side: Normal functioning in an adult with a healthy microbiome. Box 1: The balance of bodily hormones and microbiome semiochemicals communicate through the gut–brain axis to partition the flow of nutrition according to need. Box 2: Information regarding the microbial environment is accumulated in the microbiome. Right hand side: From child to adult. Box 3: This information is passed on to the neonate by the seemingly accidental process of maternal microbial inheritance. Box 4: The newly inherited microbiome of the mother helps to calibrate the immune system of the child against the antigenic environment of the mother. At the same time, the gut–brain axis starts to develop. A breakdown in these latter stages sets the scene for non-communicable disease in later life.

By contrast, the failure of the dual immune/semiochemical control system leads to a complex series of health problems normally classified by the single most troublesome variety. Thus, while coeliac disease is classed as a problem of the intestine [80], immune system problems can, nonetheless, occur anywhere around the body, along with problems of food absorption and of depression [75]. It is important to note that these non-communicable diseases are modern due only to their prevalence, as occasional examples of similar diseases can be found dating from earlier times [29]. Of course, ancient empires used heavy metals extensively and this may have functioned as a trigger to activate latent genetic susceptibilities for coeliac disease, for example [81]. Similarly, perhaps the earliest example of a non-communicable disease has been the atherosclerotic plaques detected by CT scanning of Egyptian mummies [82].

## 7. The Partition of Nutrition: The Loss of Semiochemical Control

**Figure 2:**
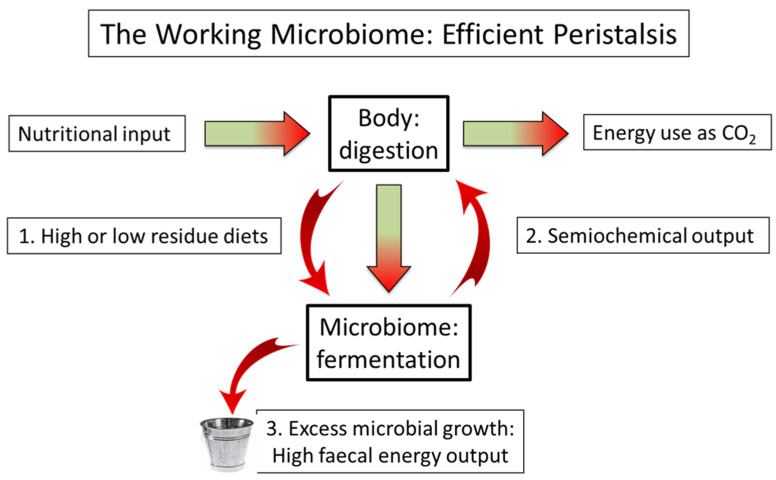
Peristalsis controlled by the gut–brain axis: Energy flow diagram.

In this section we again employ the “black box” concept to indicate that, although there are many bioactive molecules produced within the microbiome, the term semiochemical is a composite term implying that signalling molecules emitted by the microbiota strengthen the gut–brain axis. Note that this term also includes the vitamins and SCFAs that supply energy to power cells adjacent to the gut [67] as these cells are also known to be involved both with the regulation of the immune system [69] and with serotonin production [68]. Accordingly, Figure 2 illustrates the energy partition between body and microbiome consistent with our interpretation of a fully functioning microbiome, with energy outflow represented by carbon dioxide production on the one hand, and by faecal energy output via excess microbial growth on the other.

This Figure illustrates an equable partition of nutrition between bodily digestion and microbiome fermentation. Energy flow is represented by carbon dioxide on the one hand and faecal output on the another. Box 1: Following Burkitt’s observations, it seems that either high- or low-residue diets (c.f. the cattle-herding Maasai [16]) are equally conducive to high levels of gut motility and consequent absence of non-communicable disease [13]. Box 2: The flux of semiochemicals, potentially including dopamine, controls the rate of peristalsis according to the nutritional status of the individual. Box 3: Again, following Burkitt, faecal energy output will be a significant factor in the energy balance of a healthy individual [16].

**Figure 3:**
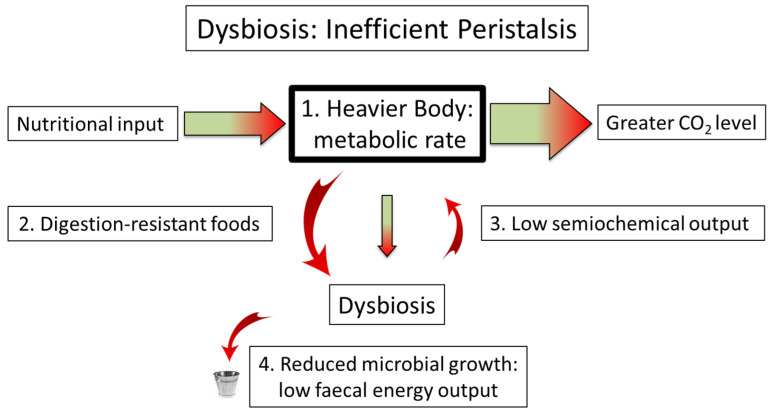
The effect of dysbiosis on energy flow and, potentially, fat distribution.

Whatever the precise mechanisms of semiochemical control, it is likely to involve peristalsis, so that its loss leads to reduced gut motility and the consequent increase in the absorption of food compared to its excretion from the body. Consequently, Figure 3 illustrates the current situation, in which the reduced output of semiochemicals follows from low microbial growth. The observations of Burkitt that the average daily faecal weight from people living in traditional societies was approximately three times that of high-income countries [13] have been supported by modern findings [83]. In a similar theme, it has been found that faeces hold 25–50% of bacteria by weight [84], and it is interesting to speculate whether the lower bacterial fractions may correspond to both a lower faecal weight and, possibly, to a higher bodyweight. This, along with an increased production of carbon dioxide, is the ultimate rationale for the obesity epidemic [16]. It is under these “dysbiotic” conditions that less readily digestible, fermentable, foodstuffs become more relevant.

This Figure illustrates the concept of increased adiposity because of reduced peristalsis. Box 1: An increase in energy stored as fat leads to a higher metabolic rate and, therefore, greater carbon dioxide outflow. In essence, microbiome-function deficiency disease (dysbiosis) causes an increase in stored fat mass until the increased metabolic rate balances the reduction in faecal energy output [16]. Box 2: A semblance of metabolic efficiency may be regained if the diet consists of a high proportion of digestion-resistant foodstuffs—dietary fibre, resistant starch, and polyphenols. Box 3: While a reduction in the quantity of semiochemical output leads to weight gain, it is possible that a drop in the mix of such agents leads to a change in the distribution of energy—visceral fat or excess growth, for example—albeit with the reduction in strength seen among 10-year-old schoolchildren [8]. Box 4: A reduction in both gut motility and microbial growth leads to a reduced faecal output, with Burkitt’s original observations confirmed more recently [83]. It is interesting to speculate whether this low output, along with a natural reluctance to work with such material, has resulted in its neglection as a cause of obesity relative to higher-profile aspects of biology and genetics [4].

Whereas the term *constipation* is normally brought to the attention of a medical professional after an actual change in bowel behaviour [85], of course no one will be able to diagnose a mere *inefficiency* of excretion without some external source of comparison. Presumably owing to the low faecal volume observed in rich countries, this aspect of the overall energy balance has not previously been considered as a cause of obesity [16].

## 8. Microbe to Market: Pro- and Pre-Biotics

Laboratory studies in mice have demonstrated the disappearance of some classes of intestinal microbes following exposure of successive generations to poor-quality, low-residue diets [86]. In turn, this has focussed attention onto the high-fibre diets originally espoused by Burkitt, a trend reinforced by the discovery of the microbial production of SCFAs [14]. Alongside these efforts, an attempt has been made to understand the development of diseases by bacterial microbiome-based prediction of glycaemic response [87], however, the method employed and conclusions reached have been challenged [88].

Following the argument that key microbes have been lost by exposure to low-residue diets, much effort has been put into either increasing the nutritional supply of live microbes, *probiotics* [89], or stimulating the activity of those already existing, *prebiotics* [90]. The combination of the two, often including oligosaccharides [91], has been described as *synbiotics* [92], while people who are interested in the mental health aspects of diet use the expression *psychobiotics* [93]. Unfortunately, while one definition of probiotics is “live microorganisms which, when administered in adequate amounts, confer a health benefit to the host” [89], it was recognised that the terms *health benefit* and *adequate amount* would be hard to define [94]. Nevertheless, the search for health improvement from live biotherapeutic microorganisms goes on [95].

Bearing in mind the comments of Brüssow [73], if the search for health-enhancing microbes is to become more focused, in general it is more likely that greater diversity will confer greater advantage [18]. Equally, the search for microbial diversity should not exclude unicellular eukaryotes, a potential missing link between the prokaryote microbiota and the multicellular Animalia [48]. In spite of these caveats, however, in certain circumstances, such as improving the outcome for preterm infants, a combination of probiotics and prebiotics can definitively be shown to be beneficial [96]. It could also be argued that the probiotic concept represents a subset of faecal microbiota transplantation and, indeed, the success of this procedure against *Clostridioides difficile* (formerly *Clostridium*) overgrowth can depend on the exact condition to be treated [97,98].

## 9. Feeding the Microbiome: Food, Fermentation, FODMAP, and Plant Polyphenols

In general, the primary difficulty with the probiotic concept is that it is hard to see why the addition of limited numbers of specific, patentable, living bacteria should make a significant contribution to the diversity of the microbiome. Indeed, a study was carried out comparing apples grown by conventional and by so-called “organic” management technologies. Analysis of the different bacterial communities suggested that, while the conventionally grown apples have greater amounts of the bacterial genera that, rightly or not, have been associated with healthier outcomes, the organically managed apples nevertheless hold a significantly greater diversity of different bacterial classes [99]. In a similar fashion, the apparent absence of any bifidobacteria, normally considered to be probiotic species [27], in the seemingly healthy Hadza [26] seems to undermine the notion that there is any value in selectively adding specific bacteria to the microbiome. As stated above, however, there is plenty of opportunity for foodstuffs in general to supply potentially valuable microbes, or their associated antigens and plasmids, even if the microbes themselves are deactivated [15]. Honey, for example, is not only a potential microbe growth-inhibitory agent, but also a staple of many hunter–gatherer societies [100]. Interestingly, although honey is indeed recognised as a source of microbes, before the recent interest in probiotics there was a concern that these bacteria were pathogens [101]. The dilemma can be resolved if the immune system successfully tackles pathogens/probiotics, while activating the gut–brain axis at the same time, in analogy with the above discussion surrounding traveller’s diarrhoea. In turn, this confirms that any such *psychobiotic-style* benefits are necessarily transitory.

Of course, many pre-modern cultures included microbes in the form of foods preserved by fermentation, of which one of the earliest examples comes from sealed vessels found in China during the seventh millennium BCE [102]. Equally, many traditional recipes survive to the present day, one example being *pozol*, made by the Maya from alkali-treated corn dough [103]. While “pre-refrigerator” people would probably have fermented foodstuffs solely in order to preserve them, recently we have started to consider such processing to be of health value in its own right. Unfortunately, of course, definitive health studies around fibre and fermentation are not easy to arrange [104].

Although dietary fibre is normally considered to be an asset, in the presence of certain non-communicable diseases, by contrast, substances requiring such microbial digestive assistance may be associated with significant gastrointestinal symptoms. In this case a FODMAP-excluding diet may be prescribed, i.e., eliminating all *f*ermentable *o*ligo-, *d*i-, and *m*ono-saccharides *a*nd *p*olyols [105] while, in addition, non-coeliac gluten sensitivity appears to be similar and also requires an exclusionary diet [106,107]. Interestingly, at least some of these dietary intolerances can be reversed by gradual reintroduction under medical supervision [108] and, although there are microbial changes throughout this process, their involvement in the progress of disease remains unclear [109]. In principle, dietary fibre-like benefits can be gained by processing starchy foods so that digestion is slowed. Studies in so-called resistant starch are ongoing [110], as are their effects on the gut microbiota, albeit covering only bacteria [111]. To our knowledge, there are no uncomfortable side-effects specifically noted with the consumption of such resistant starches.

Finally, alongside the interest in high-fibre diets, the recognition of the health benefits of the plant polyphenols [112] led initially to their description as antioxidants, based largely on their in vitro behaviour [113]. Nevertheless, the recognition of the low bioavailability of these substances [114] implies that they behave in a similar fashion to the oligosaccharides, by directly feeding the microbiome. Significantly, many phenol derivatives are themselves toxic to the body [115], implying that high concentrations in the bloodstream are unlikely to be helpful. In spite of this, however, interest in the polyphenols remains high, a recent favourite being curcumin, a major component of turmeric. Its bioavailability remains low, even with added piperine [116], and a review has cast doubt on its likelihood as a medicinal agent, primarily due to its reactivity [117]. In turn, this raises the possibility that the various components of turmeric may act directly on the intestinal microbiome, with any health benefits accruing from the production of semiochemicals stimulating the gut–brain axis. The same comments may well apply to the documented reduction in the risk of stroke and dementia associated with the consumption of polyphenol-containing tea and coffee, alongside their well-known stimulant action [118]. Finally, a recent review considers the effect of polyphenol supplementation in terms of microbial balance [119].

## 10. Microbiome-Function Deficiency Disease: Cure, Control, or Prevent?

Attempting microbiomes characterisation, we found technical variations related with sampling and statistical limitations related to cohort sizes to avoid confounding factors that do in fact influence microbial composition, such as age or sex and hormones. Some of them express in two-way interactions such as BMI, comorbidities, diet, lifestyle, medication and not only antibiotics, and even geographic region and culture; by contrast, genetic ancestry has actually minor effects [120,121,122].

We can see that changing the diet even in the short term alters not only the macronutrient intake but also the microbiota although not successfully to improve some conditions [123], because the effects will be more permanent only with long-term diets of several months or years [124]. Therefore, the microbiome–metabolome crosstalk is dependent on how the biochemistry of molecules produced by gut microbes affect the physiology. For example, fibre intake promotes SCFA and vitamin production, yogurt is itself a good probiotic source, and some polyphenols from red fruits or wine have positive effects on the host as well as for the resident microbiota, while the atherogenic molecule TMAO comes from the microbial degradation of meat proteins [121,122,125,126,127]. Additionally, even if resveratrol in wine is beneficial, smoking and alcoholism have adverse effects in digestive/absorptive microbiota by changing the stomach pH and promoting chronic gut inflammation that leads to leaky gut [128]. Even so, the boundary between the healthy and the dysbiotic gut is fuzzy, sometimes with explanations that seem ad hoc [73]. What is clearer is that a healthy microbial composition supports gut homeostasis, prevents permeability, and promotes the production of metabolites beneficial to the host [129].

Indeed, microbiota composition was found to covariate with a myriad of factors. In a Belgian study, 69 covariates were found to be associated with microbiota variation, with stool consistency showing the largest effect size, and medication explaining the largest variance and with more covariate–microbiota associations [130]; whereas in a Dutch study, 126 factors were found to influence microbiota composition, but a lower faecal chromogranin A was found to be a biomarker indicating higher microbiome diversity [131].

As the Introduction hopefully makes clear, people living in unpolluted environments do not suffer from extensive non-communicable disease, and, presumably, have significant faecal energy output. Just as importantly, Burkitt’s observations of the healthy Maasai confirm that, contrary to his own supposition, a fibre-rich diet is not in itself a decisive health-determining feature [13]. By contrast, as non-communicable disease arises from the pollution-initiated breakdown of the dual immune/semiochemical control system, so both systems must be considered in the process of disease modification. Sadly, of course, as the initial microbiome–gut dissociation takes place from earliest childhood, so a formal cure for affected individuals is impossible, but amelioration of diseases is yet possible [132,133]. Fortunately, perhaps, a recognition of the underlying cause may allow any incipient disease to be controlled prior to the development of symptoms. However, the most satisfactory outcome will be to add missing microbiome functionality at the moment of birth, thus preventing disease in the first place and, thereby, effectively future-proofing humanity to face serious challenges such as those posed by climate change.

Bearing in mind that it is diversity and equilibrium that make an individual healthy, and that many types of related organisms secrete and consume the same compounds, it is reasonable to consider a search method based on semiochemicals rather than looking for a plethora of compounds [134], or searching phylum by phylum [135,136], in faecal samples involving collection and storage problems [120], to discover whether gut microbes exist at adequate levels. Therefore, diverse types of pill-like devices bearing detectors and transmitters have been designed for numerous applications, such as: imaging, measuring pH or temperature, medication monitoring or with a drug reservoir, receiving acoustic data and transforming it to vital sign monitoring [137,138], for luminescence-based sensing using engineered bacteria [139], and to sense gas pressure, as shown most recently in [140]. Interestingly, two of the authors—DS and SJ—have recently suggested the development of an ingestible sensor, where the detector is calibrated to measure levels of semiochemicals produced in the gut lumen, potentially including dopamine [141]. Using our microbiome-function approach, it is worth stating that, in principle, the microbiome-gut–brain axis should produce semiochemicals to stimulate gut motility regardless of the type of food supplied to the animal. By contrast, in the presence of dysbiosis, with weaker gut–brain axis and/or less efficient production of semiochemicals, a less readily digested prebiotic-like substance will be required to produce a similar signal from the ingestible sensor [141]; results can then be combined with -*omic* data and theoretical models based on metabolic fluxes [129]. Significantly, however, prior microbiome–gut dissociation may mean that even an enhanced semiochemical signal may have a limited effect on disease symptoms [75]. While animals could be used in such studies, a recent observation has illustrated a difference in the outcome of trials involving genetically identical animals raised in two different laboratories. Faecal transplantation experiments showed that the difference lay in the microbiome [142], possibly indicating a wider problem with animal models for human disease.

Prevention of future human disease by modifying the microbiome of children in vulnerable populations may be a possibility. Although faecal transplantation procedure is usually employed in the intestine of an adult recipient [27,97], in principle it would be more effective if the missing microbiota could be applied directly on to the head of the new-born, thus replicating the process of maternal microbial inheritance [143]. Unfortunately, rolling back the progress of non-communicable disease will require a greater understanding of the details of microbiome function than we possess at present. Although the connection between the microbiome with the gut–brain axis is relatively easy to comprehend, its relationship to the immune system is much less clear and may include unicellular eukaryotic microbial sentinel cells [15]. Fortunately, there are people who live in relatively unpolluted parts of the world who are already cooperating with medical investigators. Such people, including the Tsimane [25] and the Hadza [26], may be able to help in the search for relevant microeukaryotes. Principles have been established for the compensation of people for the use of their knowledge in medical advances, and such principles could be applied here [144]. In the meantime, western societies could benefit from sound scientific knowledge to attempt an improvement of our general health [145], after all, Hippocrates of Kos was right in that “all disease begins in the gut” [146,147,148].

## Data Availability

Not applicable.
